# Supplementary material for: The Effect of the Varietal Type, Ripening Stage, and Growing Conditions on the Content and Profile of Sugars and Capsaicinoids in Capsicum Peppers
Source: Plants (Basel). 2023 Jan 4;12(2):231. doi: 10.3390/plants12020231 (PMC9863480; doi:10.3390/plants12020231)
Supplement: Supplementary file 1 [file plants-12-00231-s001.zip › plants-2084534-supplementary.pdf]

Supplementary Materials

Table S1. Individual and total sugars average content ± SE (g kg $^{-1}$ FW) of unripe peppers fruits of different accessions growing under organic and conventional conditions over two years.

		2016				2017			
		Fructose	Glucose	Sucrose	Total sugars	Fructose	Glucose	Sucrose	Total sugars
Conventional	*C. annuum*								
	BGV10582	9.01±0.86 d	10.43±1.17 c	0.37±0.07 a	19.81±1.97 bc	7.98±1.23 c	6.77±1.01 bc	0.87±0.11 a	15.62±2.32 ab
	BOLA	2.01±0.31 a	4.21±0.70 a	4.38±0.78 ab	10.61±1.28 a	3.98±0.59 ab	9.57±0.44 cd	10.14±2.46 c	23.70±1.60 c
	ESPELETTE	5.08±0.44 b	7.21±0.70 b	10.99±2.88 cd	23.27±2.10 bc	7.82±1.15 c	10.46±1.87 d	8.57±2.32 c	26.85±4.69 c
	GERNIKA	5.14±1.13 b	7.01±1.53 ab	12.51±3.77 d	24.65±1.13 cd	5.88±0.71 abc	8.76±1.00 bcd	10.32±2.39 c	24.95±3.73 c
	GUINDILLA	6.60±0.36 bcd	6.90±0.61 ab	2.86±0.69 ab	16.35±1.07 ab	5.91±0.29 abc	7.87±0.61 bcd	8.23±0.99 bc	22.01±1.41 bc
	JALAPEÑO	12.73±1.37 e	8.03±1.02 bc	1.86±0.41 a	22.62±2.76 bc	11.73±1.60 d	10.58±1.26 d	1.77±0.41 a	24.08±3.21 c
	PIQUILLO	6.01±0.55 bc	6.61±0.65 ab	6.99±2.12 bc	19.61±2.98 bc	3.26±0.54 a	3.34±0.62 a	4.50±0.74 ab	11.09±1.41 a
	SERRANO	15.36±2.29 e	13.97±2.25 d	0.88±0.30 a	30.21±4.38 d	11.57±0.83 d	14.99±0.84 e	3.08±0.25 a	29.64±1.81 c
	Otras ssp								
	BOL58	6.82±0.69 bcd	8.42±0.60 bc	1.41±0.12 a	16.65±1.19 ab	3.83±0.71 ab	6.37±1.12 ab	2.31±0.46 a	12.51±2.24 a
	ECU994	8.41±0.81 cd	8.79±0.67 bc	1.39±0.05 a	18.58±1.37 bc	6.45±0.87 bc	7.38±1.18 bc	0.82±0.28 a	14.65±2.21 ab
Organic	*C. annuum*								
	BGV10582	12.17±1.05 c	11.16±1.32 c	1.00±0.06 a	24.32±2.40 c	8.79±0.65 f	8.08±0.58 c	0.84±0.07 ab	17.71±1.26 de
	BOLA	1.86±0.16 a	3.82±0.63 a	11.44±1.71 d	17.11±2.07 abc	2.17±0.33 ab	2.83±0.77 a	3.60±0.87 cd	8.60±1.23 ab
	ESPELETTE	4.77±1.15 ab	6.07±1.41 ab	4.61±1.77 bc	15.45±3.39 ab	6.05±0.56 de	5.87±0.59 bc	9.83±0.56 e	21.74±0.96 e
	GERNIKA	6.63±0.58 b	7.08±1.51 abc	6.38±1.98 c	20.10±1.86 abc	1.85±0.16 a	2.00±0.12 a	1.70±0.15 abc	5.55±0.33 a
	GUINDILLA	5.24±0.55 b	5.45±0.58 ab	2.03±1.05 ab	12.72±1.34 a	7.34±0.39 ef	7.49±0.40 c	2.61±0.47 bc	17.44±0.60 de
	JALAPEÑO	12.20±1.63 c	7.67±1.91 abc	1.06±0.60 a	20.94±3.91 bc	14.88±0.71 h	14.20±0.82 d	2.94±0.28 c	32.02±1.64 f
	PIQUILLO	7.50±1.83 b	6.76±2.58 abc	5.49±3.65 bc	19.75±3.55 abc	3.87±0.80 bc	3.55±0.83 ab	5.46±1.69 d	12.87±1.71 bc
	SERRANO	19.93±1.93 d	17.11±2.03 d	0.59±0.10 a	37.64±3.93 d	12.90±0.92 g	15.52±1.08 d	3.32±0.34 c	31.74±2.31 f
	Otras ssp								
	BOL58	6.31±0.24 b	8.26±0.70 bc	1.97±0.22 ab	16.54±0.84 abc	4.83±0.51 cd	8.03±1.08 c	2.50±0.76 bc	15.36±2.16 cd
	ECU994	6.84±0.14 b	7.62±0.53 abc	1.07±0.04 a	15.53±0.69 ab	5.89±0.75 de	6.28±0.86 c	0.00±0.00 a	12.16±1.61 bc

Means within columns for each growing system separated by different letters are significantly different according to the Student-Newman-Keuls multiple range test (p = 0.05).

Table S2. Individual and total sugars average content ± SE (g kg $^{-1}$ FW) of fully ripe peppers fruits of different accessions growing under organic and conventional conditions over two years.

		2016						2017					
		Fructose		Glucose		Total sugars		Fructose		Glucose		Total sugars	
Conventional	*C. annuum*												
	BGV10582	16.53±0.69	a	11.87±0.66	a	28.40±1.26	ab	8.67±1.06	ab	7.46±0.86	ab	16.13±1.92	ab
	BOLA	12.69±2.32	a	8.93±1.86	a	21.61±4.18	a	11.94±1.48	b	9.77±0.98	bc	21.70±2.41	b
	ESPELETE	27.77±1.86	de	20.61±2.49	b	48.38±4.20	d	23.15±1.35	c	20.58±1.44	f	43.74±2.79	c
	GERNIKA	34.37±3.06	f	31.14±3.65	c	65.51±6.64	e	20.91±2.02	c	18.29±1.28	ef	39.20±3.28	c
	GUINDILLA	21.71±0.70	bc	13.44±0.43	a	35.15±1.08	bc	20.75±1.16	c	19.47±1.17	ef	40.22±2.33	c
	JALAPEÑO	24.79±2.07	cd	18.65±1.32	b	43.44±3.28	cd	23.54±1.41	c	18.95±1.04	ef	42.49±2.44	c
	PIQUILLO	12.72±1.73	a	10.25±1.60	a	22.97±3.31	a	21.23±3.91	c	14.99±2.86	de	36.22±6.76	c
	SERRANO	29.85±0.82	ef	38.49±1.40	d	68.35±2.17	e	19.46±2.33	c	16.40±2.31	def	35.86±4.64	c
	Otras ssp												
	BOL58	34.40±1.10	f	27.05±1.10	c	61.45±1.82	e	5.23±0.39	a	4.81±0.40	a	10.04±0.79	a
	ECU994	17.45±0.69	ab	12.94±0.74	a	30.39±1.39	ab	13.42±2.36	b	12.25±2.06	cd	25.67±4.42	b
Organic	*C. annuum*												
	BGV10582	18.69±1.45	b	18.23±2.05	bc	36.92±3.47	b	13.83±1.50	b	11.93±1.32	bc	25.76±2.82	b
	BOLA	19.42±1.04	bc	14.70±1.01	ab	34.11±2.03	ab	17.97±0.79	bc	15.27±0.96	c	33.24±1.57	b
	ESPELETTE	18.57±4.07	ab	14.76±3.09	ab	33.33±7.14	ab	18.97±2.00	bc	13.83±1.79	c	32.80±3.77	b
	GERNIKA	29.23±1.95	def	28.25±2.34	d	57.49±4.28	de	8.20±0.88	a	7.05±0.71	ab	15.24±1.59	a
	GUINDILLA	24.45±0.24	cd	22.86±0.58	c	47.32±0.76	c	16.92±2.45	bc	15.28±2.34	c	32.20±4.80	b
	JALAPEÑO	33.50±1.11	f	21.53±0.91	c	55.03±2.00	cde	28.03±1.64	d	25.24±1.56	d	53.26±3.20	c
	PIQUILLO	13.50±2.20	a	11.25±2.25	a	24.75±4.43	a	19.45±2.72	c	15.65±2.51	c	35.10±5.23	b
	SERRANO	30.68±0.98	ef	31.78±0.77	d	62.46±1.74	e	17.35±3.13	bc	15.07±3.13	c	32.41±6.24	b
	Otras ssp												
	BOL58	28.34±0.54	de	21.12±0.66	c	49.46±1.02	cd	4.65±0.65	a	4.08±0.52	a	8.73±1.17	a
	ECU994	16.47±1.26	ab	12.16±0.99	a	28.62±2.24	ab	14.98±0.61	bc	12.32±0.42	c	27.61±1.02	b

Means within rows for each growing system separated by different letters are significantly different according to the Student-Newman-Keuls multiple range test (p = 0.05).

Table S3. Individual and total capsacinoids average content ± SE (mg kg $^{-1}$ FW) of unripe ripe peppers fruits of different accessions growing under organic and conventional conditions over two years.

		2016								2017							
		NDHC		C		DHC		TOTAL CS		NDHC		C		DHC		TOTAL CS	
Conventional	*C. annuum*																
	ESPELETTE	1.96±0.41	b	33.07±5.43	bc	16.72±2.90	cd	51.74±8.50	bc	1.08±0.12	abc	22.87±	ab	8.88±1.53	ab	32.83±5.63	a
	GERNIKA	0.00±0.00	a	5.36±1.89	a	1.71±1.01	ab	7.07±2.88	a	0.00±0.00	a	2.26±	a	0.00±0.00	a	2.26±0.41	a
	GUINDILLA	2.85±0.56	b	41.68±6.32	c	24.13±3.55	d	68.66±10.26	c	2.06±0.20	c	27.10±	ab	15.89±2.47	b	45.05±6.17	ab
	JALAPEÑO	2.96±0.48	b	11.00±2.39	ab	10.76±1.97	bc	24.72±4.72	ab	0.39±0.08	ab	2.21±	a	1.38±0.30	a	3.99±0.80	a
	SERRANO	14.99±1.20	c	79.80±7.06	d	59.87±5.19	e	154.66±13.40	d	15.63±1.05	e	77.66±	c	62.61±4.06	e	155.90±11.25	c
	Otras ssp																
	BOL58	0.00±0.00	a	2.30±0.28	a	0.00±0.00	a	2.30±0.28	a	9.99±1.00	d	52.45±	bc	29.56±2.32	c	92.00±5.79	b
	ECU994	2.23±0.18	b	381.18±24.54	e	78.39±3.69	f	461.79±27.62	e	2.03±0.31	bc	258.60±	d	49.69±7.19	d	310.31±43.91	d
Organic	*C. annuum*																
	ESPELETTE	0.86±0.09	b	23.30±2.36	bc	11.51±1.30	b	35.67±3.63	b	1.59±0.47	a	23.64±	ab	12.21±3.77	a	37.44±9.40	a
	GERNIKA	0.09±0.09	a	5.23±2.46	a	1.74±0.76	a	7.06±3.30	a	0.00±0.00	a	2.02±	a	0.00±0.00	a	2.02±0.33	a
	GUINDILLA	1.24±0.18	bc	27.70±4.07	c	13.45±1.89	b	42.39±6.10	b	1.56±0.38	a	24.94±	ab	11.38±1.65	a	37.87±4.87	a
	JALAPEÑO	2.47±0.32	d	12.31±1.26	ab	13.03±1.33	b	27.81±2.88	b	0.00±0.00	a	0.70±	a	0.00±0.00	a	0.70±0.06	a
	SERRANO	14.76±0.35	e	66.04±2.92	d	55.74±2.22	c	136.55±5.09	c	14.81±0.95	b	74.61±	c	66.55±6.80	c	155.98±11.22	c
	Otras ssp																
	BOL58	0.00±0.00	a	2.05±0.16	a	0.00±0.00	a	2.05±0.16	a	13.61±1.62	b	49.32±	bc	38.00±3.64	b	100.93±8.30	b
	ECU994	1.80±0.13	c	346.80±10.53	e	66.69±1.73	d	415.29±12.14	d	1.79±0.25	a	261.55±	d	45.69±6.03	b	309.03±42.59	d

Means within rows for each growing system separated by different letters are significantly different according to the Student-Newman-Keuls multiple range test (p = 0.05).

Table S4. Individual and total capsacinoids average content ± SE (g kg $^{-1}$ FW) of fully ripe peppers fruits of different accessions growing under organic and conventional conditions over two years.

		2016				2017			
		NDHC	C	DHC	CS totales	NDHC	C	DHC	CS totales
Conventional	*C. annuum*								
	ESPELETTE	3.99±1.32 bc	37.69±9.11 a	20.34±5.08 b	62.02±15.16 ab	2.72±0.54 ab	30.08±5.74 b	16.31±3.13 bc	49.11±9.30 b
	GERNIKA	1.43±0.37 a	12.78±4.47 a	2.43±1.73 a	16.64±6.09 a	1.10±0.28 a	7.17±1.83 ab	0.74±0.42 a	9.02±2.14 a
	GUINDILLA	5.42±0.23 cd	62.74±2.84 ab	43.34±2.84 c	111.51±5.35 b	2.51±0.31 ab	27.05±3.39 ab	22.98±9.94 cd	52.54±8.70 b
	JALAPEÑO	4.27±1.19 bc	17.14±4.74 a	13.13±3.80 ab	34.53±9.67 a	1.05±0.20 a	5.74±0.93 a	3.46±0.81 ab	10.25±1.85 ab
	SERRANO	18.92±0.91 e	112.09±4.33 b	88.09±3.17 d	219.09±8.31 c	15.36±2.12 d	73.68±9.17 c	63.37±7.88 e	152.41±19.13 d
	Otras ssp								
	BOL58	2.66±0.25 ab	8.52±1.07 a	2.04±0.36 a	13.21±1.58 a	12.27±1.12 c	56.00±4.93 c	34.32±3.55 d	102.59±9.35 c
	ECU994	6.55±0.23 d	574.21±57.19 c	109.78±8.41 e	690.55±65.67 d	4.37±0.41 b	368.84±16.8	66.21±3.31 e	438.94±20.3 e
Organic	*C. annuum*								
	ESPELETTE	4.63±0.77 bc	44.43±3.29 b	29.28±1.59 b	78.35±3.96 b	2.28±0.92 ab	18.18±6.49 a	11.00±4.17 b	31.46±11.55 a
	GERNIKA	2.13±0.18 a	7.69±0.90 a	0.74±0.52 a	10.55±1.57 a	1.45±0.09 a	4.54±0.60 a	0.00±0.00 a	5.99±0.68 a
	GUINDILLA	7.52±0.57 d	59.59±7.29 bc	48.79±5.51 c	115.90±13.16 c	4.95±0.73 b	54.98±5.23 b	31.24±4.01 c	91.17±9.68 b
	JALAPEÑO	3.19±0.71 ab	10.66±2.94 a	8.67±4.02 a	22.52±7.36 a	0.51±0.15 a	3.01±0.77 a	1.09±0.56 ab	4.61±1.48 a
	SERRANO	13.29±1.54 e	66.83±6.58 c	57.81±5.60 c	137.93±13.64 c	11.17±0.65 c	53.03±3.00 b	49.21±2.58 d	113.41±6.15 b
	Otras ssp								
	BOL58	3.45±0.49 ab	7.51±0.81 a	1.95±0.48 a	12.91±0.84 a	18.26±2.65 d	48.11±6.37 b	41.94±5.92 cd	108.30±14.87 b
	ECU994	6.24±0.23 cd	621.03±14.04 d	113.97±1.89 d	741.24±15.96 d	4.89±0.30 b	466.10±23.04 c	86.96±4.58 e	557.95±27.91 c

Means within rows for each growing system separated by different letters are significantly different according to the Student-Newman-Keuls multiple range test (p = 0.05).

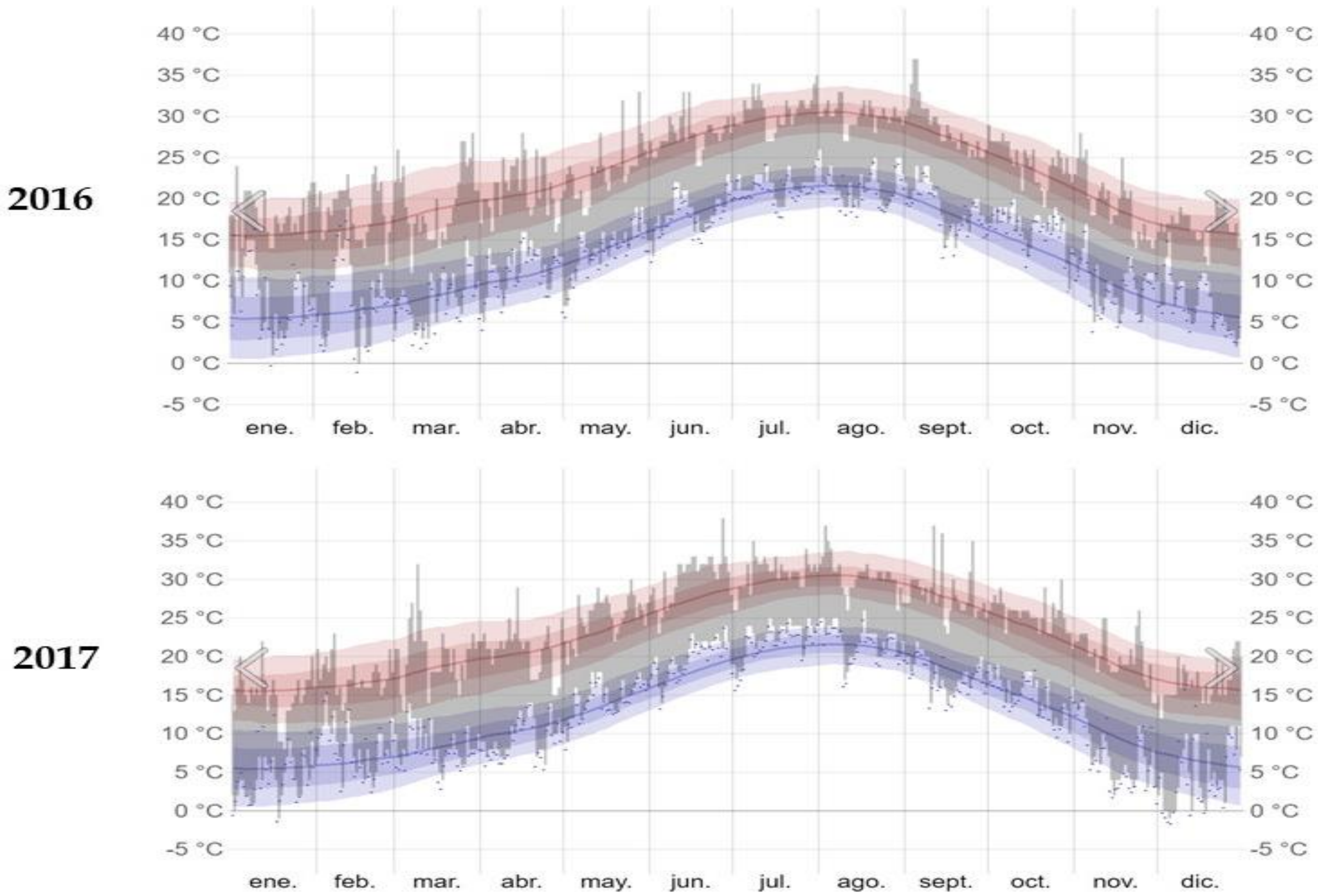

Figure S1. Daily evolution of temperatures in years 2016 and 2017 in the area of Sagunto, where the experiments were performed. Grey bars represent, day by day, the range of temperatures (from minimum to maximum temperature) in each year. The red line represents, as a reference, the mean maximum temperature for each day, while the blue line represents the mean minimum temperature for each day (based on records since 1970). Red and pale red bands represent percentiles 25-75% and 10-90% for max temperatures, respectively, while blue and pale blue bands represent percentiles 25-75% and 10-90% for min temperatures (adapted from https://es.weatherspark.com/y/42639/Clima-promedio-en-Sagunto-Espa%C3%B1a-durante-todo-el-a%C3%B1o).
